# Lin28A/CENPE Promoting the Proliferation and Chemoresistance of Acute Myeloid Leukemia

**DOI:** 10.3389/fonc.2021.763232

**Published:** 2021-11-12

**Authors:** Mingyue Shi, Junwei Niu, Xiaona Niu, Honggang Guo, Yanliang Bai, Jie Shi, Weiya Li, Kai Sun, Yuqing Chen, Fengmin Shao

**Affiliations:** ^1^ Department of Hematology, Zhengzhou University People’s Hospital and Henan Provincial People’s Hospital, Zhengzhou, China; ^2^ Department of Nephrology, Henan Provincial Key Laboratory of Kidney Disease and Immunology, Zhengzhou University People’s Hospital and Henan Provincial People’s Hospital, Zhengzhou, China

**Keywords:** CENPE, LIN28A, AML, chemoresistance, cell cycle

## Abstract

The prognosis of chemoresistant acute myeloid leukemia (AML) is still poor, mainly owing to the sustained proliferation ability of leukemic cells, while the microtubules have a major role in sustaining the continuity of cell cycle. In the present study, we have identified CENPE, a microtubular kinesin-like motor protein that is highly expressed in the peripheral blood of patients with chemoresistant AML. In our *in vitro* studies, knockdown of CENPE expression resulted in the suppression of proliferation of myeloid leukemia cells and reversal of cytarabine (Ara-C) chemoresistance. Furthermore, Lin28A, one of the RNA-binding oncogene proteins that increase cell proliferation and invasion and contribute to unfavorable treatment responses in certain malignancies, was found to be remarkably correlated with CENPE expression in chemoresistance AML. Overexpression of LIN28A promoted the proliferation and Ara-C chemoresistance of leukemic cells. RIP assay, RNA pull-down, and dual luciferase reporter analyses indicated that LIN28A bound specifically to the promoter region GGAGA of CENPE. In addition, the impacts of LIN28A on cell growth, apoptosis, cell cycle progression, and Ara-C chemoresistance were reverted by the knockdown of CENPE. Hence, Lin28A/CENPE has enhanced the proliferation and chemoresistance of AML, and therefore, it could be a prospective candidate for AML treatment.

## Introduction

The prognosis of chemoresistant acute myeloid leukemia (AML) remains poor due to the sustained proliferative capacity of leukemic cells (1–3). The dysregulated cell cycle could induce raised proliferation, which predisposes leukemic cells to gain mutations and may privilege chemoresistant leukemic transformation (4–6). Cell cycle-specific agent cytarabine (Ara-C) and cell cycle-nonspecific agents anthracycline chemotherapeutics are the standard treatment of AML in both induction and consolidation therapies, but still a proportion of patients present intrinsic or acquired chemotherapy resistance (7, 8). Thus, there is an urgent need for new targets and therapeutic approaches to treat chemoresistant AML. Cell cycle checkpoint, including microtubules, is critical in the maintenance of a continuous cell cycle. Targeting cell cycle checkpoints has showed promising results in preclinical models and provides a potential combination therapy for AML patients (3, 5, 9).

Centromere protein E (CENPE), a microtubule kinesin, localizes to unlinked kinesins during mitosis and slides monomeric chromosomes toward the spindle equator using end-directed microtubule movement (10). The upregulated CENPE has been found to be involved in the tumorigenesis of breast cancer, prostate cancer, neuroblastoma, etc., and CENPE deletion could lead to the apoptosis of tumor cells (11–14). A most recent study demonstrated that in medulloblastoma cells, CENPE depletion triggered the endogenous DNA damaging, which activated TP53 or TP73 and cell death signaling pathways (15). In a research of 1,195 non-small cell lung cancer (NSCLC) patients’ samples, CENPE was revealed to be highly expressed and patients with strong CENPE expression had a relatively low overall survival rate (16). In prostate cancer, CENPE expression could be activated by LSD through binding to the promoter region (13). To understand the mechanism of CENPE depletion in tumor cell growth, an *in vitro* study has further identified that the overexpressed FOXM1 could facilitate CENPE expression and lung cancer cell proliferation by specifically binding to the CENPE promoter region (17).

In leukemia, attention has already been devoted to antimitotic agents. For example, in HL-60 cells, the antimitotic agent HKL-1 was found to evoke mitotic catastrophes by downregulating the mitotic stage-specific kinase CENPE and downregulating Bcl-2 (18). Moreover, an anti-mitogenic agent GSK923295A, capable of inhibiting CENPE motility activity, exhibited substantial remission-inducing antileukemia activities towards acute lymphoblastic leukemia (ALL) xenografts (19). In 38,410 cells from aspirates of AML patients and healthy volunteers, single-cell RNA-seq and genotyping were performed and CENPE was found to be related to minimal residual diseases (MRD) >2-fold standard deviation of all residuals (20). However, the mechanism of CENPE in AML progression and chemoresistance is rarely studied.

RNA binding proteins (RBPs) are key modulators of cancers and mRNAs (21, 22). Previous studies have explored the effect and molecular mechanisms of the RBPs LIN28A in the development of various tumors and revealed the underlying role of LIN28A on cell cycle-related mRNAs (23–26). Lin28 has been found to enhance the growth of dental pulp cells by upregulating the cyclin-dependent proteins and by interacting with the let-7a/IGF2BP2 pathway (23). In epithelial tumors, LIN28A promoted cell cycle procession by moderating the expression of CDK2, Cyclin D1, and CDC25A (26). Highly expressed LIN28A can serve as a potential oncogenic factor that contributes to the tumorigenesis, development, and migration of ovarian, breast, liver, and colon cancers (27–33). Mechanism-wise, LIN28A can modulate the translation of its targeted mRNA and restrain let-7 expression in the posttranscriptional level, which both depend on the LIN28A protein’s RNA-binding motif (34–42). For example, in a study on colorectal cancer, LIN28A was found to promote the development and progression of disease by regulating the expression of the mRNA GEFT (38). Moreover, LIN28A has been confirmed to have the capacity to stabilize and modulate the expression of various mRNAs, including YB-packaged mRNA, RAN, and HSBP1 mRNA in tumors (40–42). More interestingly, it has been shown that LIN28A participated in regulating the differentiation and cell cycle progression of AML cells (43). However, the mechanism of LIN28A in AML progression and chemoresistance is not definitively understood.

In our study, we found that CENPE was overexpressed in patients with chemoresistant AML. Furthermore, Lin28A was found to be remarkably correlated with CENPE expression in chemoresistance AML. Knockdown of CENPE expression led to the suppression of growth of myeloid leukemia cells and reversal of Ara-C chemoresistance. Overexpression of LIN28A promoted the growth and Ara-C of leukemic cells by specifically binding to the promoter region GGAGA of CENPE, while knockdown of CENPE reverted this influence. Our findings indicated that Lin28A may have a pivotal role in AML tumorigenesis and chemoresistance by modulating CENPE, and that targeting Lin28A/CENPE could be a potential effective treatment or combined chemotherapy regimen for chemoresistant AML patients.

## Materials and Methods

### Clinical Samples

Peripheral blood samples of three refractory/relapsed AML patients (R/R-AML, relapsed/refractory AML patients who failed to achieve complete remission/CR after two courses of induction chemotherapy), three refractory secondary AML patients (S-AML-, MDS-, or MPN-derived AML patients did not reach CR after two rounds of induction chemotherapy), four *de novo* AML patients (AML, CR after standard “3+7” induction chemotherapy), and three healthy controls (HC) were collected in Henan Provincial People’s Hospital. Permission of this study was obtained from the Ethics Committee of Henan Provincial People’s Hospital, and written informative consent was granted to all subjects.

### Cell Separation and RNA Extraction

Peripheral blood mononuclear cells (PBMCs) from all individuals were collected and separated by density centrifugation (Ficoll-Hypaque). All specimens were obtained from EDTA peripheral blood in 4 h and then preserved at −80°C. Total PBMC RNA was obtained by TRIzol reagent (ThermoFisher Scientific) following directions of the manufacturer. Add 0.5 ml of Trizol, RT 2–3 min. Add 0.25 ml of chloroform and shake vigorously for 20–30 s, RT 2–3 min. Then, centrifuge for 10 min at 12,000 rpm at 4°C. Carefully transfer the supernatant to another tube, add 0.5 ml of isopropanol, mix, and put in RT 10 min. Then, centrifuge for 10 min at 4°C at 12,000 rpm. Wash with 70% EtOH and air-dry the pellet. Using 50 μl of DEPC-H_2_O, dissolve the pellet. Measure OD260. Store at −80°C.

### RNA-seq and Bioinformatic Analysis

Nanodrop was applied to quantify the total RNA samples. Illumina kits were used to prepare the RNA-seq library. Ultimately, after quantifying and qualifying the RNA-seq libraries, the sequencing is detected by Illumina Hiseq 4000. Differentially expressed genes (DEGs) were screened for adjusted *p* < 0.05 and fold change ≥2. DEGs between each of the two groups were presented by scatter plot, volcano plot, and hierarchical clustering. To discover the potential underlying biological procedures and pathways in R/R-AML, S-AML, and *de novo* AML, we conducted GO and KEGG pathway analysis.

### Downloaded TCGA and GEO RNA-seq Data

Whole blood RNA-seq dataset of Recurrence-AML (R-AML) was downloaded from TCGA (151 cases) and primary AML dataset was downloaded from GEO (7 cases). The DEGs between R-AML and primary AML samples were identified based on screening criteria: |log2FC| ≥ 1 and *p ≤* 0.05. The clinical data of R-AML patients from TCGA were extracted. The expression profiles of CENPE were extracted and compared in R-AML and primary AML groups. X-tile software was used to calculate the cutoff values of CENPE in R-AML patients, and survival analysis was conducted in R-AML patients with CENPE high expression and R-AML patients with CENPE low expression.

### Cell Culture and Transfection

K562 and THP-1 cell lines were obtained from the American Type Culture Collection (ATCC). Cells were incubated in RPMI 1640 media (Sigma Aldrich, USA) with 1% penicillin/streptomycin (37°C, 5% CO_2_) and 10% fetal bovine serum (Gibco, USA). 293T cells were cultivated in DMEM media (Sigma Aldrich, USA). Search the gene sequences of CENPE on the NCBI GENE bank database, and design RNA interference sequences according to the design principles. Small interference RNA (siRNA)-directed CENPE and the negative control (NC) were made by Wuzhou Kangjian Biological Technology Co., Ltd. (Tianjin, China). The LIN28A expression plasmid and NC plasmid were purchased from Wuhan GeneCreate Biological Engineering Co., Ltd. (Wuhan, China) and transfected into K562 and THP-1 cells. Transfections were carried out in six-well plates applying Lipofectamine 3000 (Thermo Fisher Scientific, Inc.). The sequences of the siRNAs are as follows: CENPE#1: AGGCTACAATGGTACTATATT, CENPE#2: CCAAAGATTCAGCACTACTAA, Lin28A#1: CTTTCGAGAGGAAGAAGAAGA, Lin28A#2: GAGTAAGCTGCACATGGAAGG.

### Cell Proliferative Ability Analysis

Use Cell Counting Kit-8 (CCK-8, Solarbio) to observe the *in vitro* cell proliferation after transfection. In the CCK8 assay, 12 h post-transfection, 100 μl of cell suspension (about 5,000 cells/well) was transferred into a 96-well plate and then cultured at 37°C, in 5% CO_2_. Add to each well of the plate 10 μl of CCK-8 solution. Incubate the plate for 1–4 h. Thereafter, the absorbance was evaluated at 450 nm (OD450) using an automatic microplate reader. The experiment was performed at 12 h, 24 h, 48 h, and 72 h to create a cell growth curve.

### Actinomycin D Assay to Analyze mRNA Stability

Actinomycin D (ActD) was added to si-NC or si-LIN28A transfected K562 and THP-1 cells 48 h after transfection. CENPE mRNA expression was measured by RT-qPCR after 0, 2, 4, and 6 h of ActD treatment.

### Drug Treatment and IC50 Calculation

IC50 value is the drug concentration value corresponding to the cell survival rate of 50%. IC50 values were examined by the CCK-8 assay (Solarbio). To calculate K562 and THP-1 IC50 values, cells were treated with Ara-C at concentrations of 0.125 µM, 0.25 µM, 0.5 µM, 1 µM, 2 µM, 4 µM, and 8 µM at 37°C with 5% CO_2_. After 48 h, under light-proof conditions, 10 μl of CCK-8 solvent was pipetted to every well and placed at 37°C for 2 h. The absorbance was evaluated at OD450. Calculate the cell survival rates.

### Cell Apoptosis Analysis

Cells were treated either with or not with Ara-C for 48 h before collection, the cell culture supernatant was discarded, and then the cells were collected. The cells were washed twice with the phosphate buffered saline (PBS, Servicebio) and 500 µl of 1× binding buffer was added. Continue to add 5 μl each of Annexin V-FITC and PI staining solution (Solarbio) to the tube, incubate for 15 min in the dark (room temperature), and detect apoptosis by flow cytometry within 1 h.

### Cell Cycle Analysis

Cells were starved before transfection for 24 h and confirmed that most of the cells were in G0/G1 phase. Afterwards, cells were transfected with si-NC or si-CENPE, and the effect of CENPE interference on cell cycle was examined 48 h later. Wash the cells twice with PBS solution, centrifuge them, and discard the supernatant. Add 70% alcohol (pre-cooled) to 2 ml of the EP tube and centrifuge at 4°C for 30 min. The cells were collected, washed once with PBS, and centrifuged; RNase A was added; and the mixture was incubated 30 min at 37°C and then centrifuged. Continue to add 5 μg/ml of PI staining solution (Solarbio, China), place at room temperature in the dark for 15 min, and detect the cell cycle using flow cytometry.

### RT-PCR Measurement

K562 and THP-1 cell lines with or without targeted genes knocked down were collected to extracted total RNA. cDNA was synthesized applying a Bio-Rad iScript cDNA Synthesis Kit. RT-PCR was conducted with SYBR Green reaction system (12 μl). PCR primers were synthesized by Wuhan GeneCreate Biological Engineering Co., Ltd. Transfer the diluted (20 μl cDNA + 280 μl ddH2O) cDNA to an 8-strip PCR tube. Use an electric multi-channel pipette to transfer to a 384-well plate (three replicates for each test sample). Mix 2×SYBR Green Mix (ThermoFisher Scientific, USA) with primers. Centrifuge the sealing plate and test on the machine. The qPCR process is done on a CFX96 real-time system. The relative levels of mRNAs were measured using the 2^−ΔΔCq^ method. The sequences were as follows: CENPE: Forward GATGACCTAGCAACTACACAGTC, Reverse AAAGCACCCAAACTCGAATCA; LIN28A: Forward GGTGGACGTCTTTGTGCACCAGAG, Reverse CGCTCACTCCCAATACAGAACACAC; β-actin: Forward ACCAACTGGGACGACATGGAG, Reverse GTGAGGATCTTCATGAGGTAGTC.

### Western Blot Analysis

Collect 1 × 10^6^ each of K562 and THP-1 cells, wash the cells three times, then add RIPA protein lysis solution, and place on ice to lyse for 10 min. Take a small amount of protein solution for BCA protein concentration assay (Sangon Biotech, Shanghai, China). Subsequently, 50 μg of protein samples was added to the loading wells of each lane in an SDS-PAGE gel; after electrophoresis at 70 V for 25 min, switch to 120 V and continue electrophoresis for 1 h. The proteins were then moved to PVDF membranes. Block the membranes with 5% BSA (Solarbio) at room temperature for 2 h. Wash with TBST solution and add primary antibodies (anti-CENPE, anti-LIN28A, and anti-β-actin), and then incubate at 4°C overnight. Wash the PVDF membranes and then place in HRP-labeled secondary antibodies for 1.5 h, at 37°C. After sufficient washing with TBST solution, ECL chemiluminescence was performed and protein levels were analyzed.

### RIP-qPCR to Identify the Targeting Relationship Between LIN28A and CENPE

After 48 h transfection of LIN28A and si-CENPE, the K562 cells were collected, lysed, and stored at −80°C. In transfected (after 48 h) or un-transfected K562 cells, RIP Kit (Millipore) with IgG (Abcam, Cambridge, MA, USA) or LIN28A antibody (Abcam) was used to assess the binding potential of LIN28A to CENPE. The level of CENPE mRNA that was enriched by IgG or LIN28A antibodies was measured by RT-qPCR.

### RNA Pull-Down

The interaction between CENPE mRNA 3’UTR and LIN28A protein was analyzed using the RNA Pull-Down kit (Thermo Scientific). Lyse the cells with IP Lysis Buffer. Biotin-labeled CENPE mRNA 3’UTR probes for the sense or antisense strands of LIN28A were prepared. RNA pull-down experiments were performed in the whole cell lysates of K562 cells with a magnetic RNA pull-down kit. LIN28A protein levels that were pulled down by biotin-labeled transcripts were detected by Western blot.

### Dual Luciferase Report Analysis

The wild-type CENPE (CENPE Wt) 3’UTR sequence containing a LIN28A binding site was constructed onto the pGL3-Basic vector to build the CENPE Wt reporter vector. The CENPE 3’UTR and LIN28A binding site in CENPE Wt was mutated to construct the CENPE mutation (CENPE Mut) reporter vector. The LIN28A overexpression plasmid (LIN28A) and empty plasmid (Vector) were provided by Wuhan GeneCreate Biological Engineering Co., Ltd. In K562 cells, CENPE Wt and CENPE Mut were transfected with the groups of CENPE Wt+Vector, CENPE Wt+LIN28A, CENPE Mut+Vector, and CENPE Mut+LIN28A, respectively. After 48 h of cell transfection, the change of luciferase activity was detected by luciferase activity assay kit (Promega).

### Statistical Analysis

All experiments were independently repeated three times. Differences between two groups were analyzed by *t*-test, and one-way ANOVA was applied to analyze differences between multiple groups. Experimental data were analyzed using GraphPad prism 7.0 software and shown in Mean ± SEM. Pearson correlation analysis was performed to analyze correlations, and *p* < 0.05 was thought as a significant difference (SPSS22.0).

## Results

### Mitosis Cell Cycle-Related Gene CENPE Was Upregulated in Chemoresistance AML Patients

**Figure 1:**
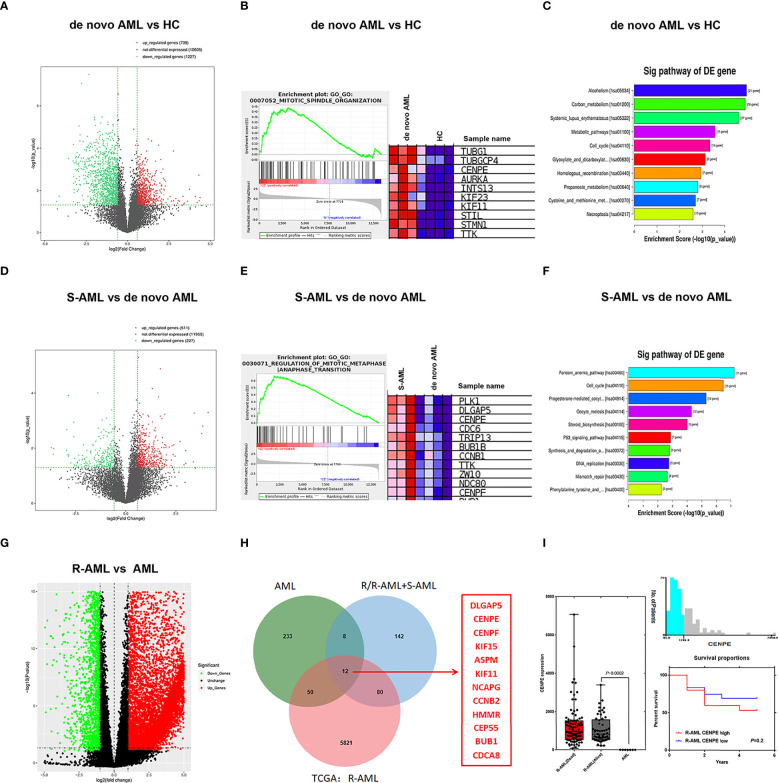
Mitosis-related gene CENPE was highly expressed in chemoresistance AML patients. **(A)** DEGs in *de novo* AML patients compared with HC. **(B)** GSEA enrichment plots of DEGs of GO biological processes were predominantly engaged in mitotic spindle organization (GO:0007052) in *de novo* AML patients compared with HC. **(C)** Upregulated DEGs enriched KEGG pathways in *de novo* AML patients compared with HC. **(D)** DEGs in S-AML versus *de novo* AML patients. **(E)** GSEA enrichment plots of DEGs of GO biological processes were predominantly engaged in regulation of mitotic metaphase/anaphase transition (GO:0030071) in S-AML versus *de novo* AML patients. **(F)** Upregulated DEGs enriched KEGG pathways in S-AML versus *de novo* AML patients. **(G)** The DEGs in R-AML from TCGA versus primary AML from GEO. **(H)** Twelve targeted upregulated DEGs among R/R-AML, S-AML, R-AML, and primary/*de novo* AML patients. **(I)** CENPE in R-AML was significantly higher than that of primary AML patients. X-tile software calculated the cutoff values of CENPE in R-AML patients, and survival analysis was conducted in R-AML patients with CENPE high expression and R-AML patients with CENPE low expression.

In the present study, RNA-seq results indicated that 1,017 genes (303 upregulated and 714 downregulated) were observed in patients with *de novo* AML in comparison to HC (**Figure 1A**). A total of 329 DEGs were acquired (202 upregulated and 127 downregulated) in chemoresistance S-AML patients compared with *de novo* AML patients (**Figure 1D**). Among S-AML samples and *de novo* AML samples, Gene Set Enrichment Analysis (GSEA) enrichment plots of DEGs of GO biological processes were predominantly engaged in mitotic spindle organization (GO:0007052) and regulation of mitotic metaphase/anaphase transition (GO:0030071) (**Figures 1B, E**). CENPE gene was in the top five upregulated DEGs (**Figures 1B, E**). In the KEGG Pathway profiling, the majority of the upregulated DEGs were as well enriched in the cell cycle pathway (hsa04110) (**Figures 1C, F**). Moreover, as to identify our hypothesis, the DEGs between R-AML from TCGA and primary AML from GEO were analyzed. When |log2FC| ≥ 1 and *p ≤* 0.05, a total of 7,957 DEGs were identified (5,964 upregulated and 1,993 downregulated) (**Figure 1G**). In order to identify the key upregulated genes in chemoresistance AML, we performed a Venn diagram analysis of the upregulated DEGs among R/R-AML, S-AML, R-AML, and primary/*de novo* AML patients, and the result revealed a total of 12 overlapping genes: CENPE, ASPM, CENPF, DLGAP5, KIF15, HMMR, BUB1B, KIF11, CEP55, NCAPG2, CCNB2, and CDCA8 (**Figure 1H**). The 12 upregulated genes were all upregulated in AML patients with relapsed and chemoresistance disease. Among the 12 overlapping genes, the *p*-value and log2 fold change of CENPE were the most significant (**Table 1**). Combined with the GSEA analysis results of DEGs in AML patients, we selected CENPE, which was enriched in the mitotic spindle organization (GO:0007052) and regulation of mitotic metaphase/anaphase transition (GO:0030071) (**Figures 1B, E**) for further study (**Figure 1E**). Moreover, we found that CENPE expression was considerably increased in R-AML compared to primary AML (**Figure 1I**). It is worth noting that the expression of CENPE in the R-AML patients ended with dead was slightly higher than that in the alive patients (**Figure 1I**). We applied X-tile software to calculate the cutoff values of CENPE in R-AML patients and divided R-AML patients into a CENPE high-expression group and a CENPE low-expression group according to the cutoff values. Although a relatively shorter survival time could be seen in the CENPE high-expression group, however, the difference between the two groups was not statistically significant (**Figure 1I**).

**Table 1:** The expression profiles of 12 upregulated overlapping genes in AML samples.

	baseMean	log2FoldChange	lfcSE	stat	pvalue	padj
CENPE	1049.802	5.191935	0.425723	12.19556	3.28E-34	1.88E-32
ASPM	1919.416	4.978494	0.427757	11.6386	2.62E-31	1.26E-29
CENPF	3083.29	4.450087	0.375638	11.84674	2.24E-32	1.16E-30
DLGAP5	570.4089	2.538413	0.434018	5.848632	4.96E-09	2.46E-08
KIF15	746.446	2.504048	0.361179	6.932982	4.12E-12	3.11E-11
HMMR	660.9024	2.39525	0.401993	5.958438	2.55E-09	1.32E-08
BUB1B	1155.31	2.240073	0.306405	7.310828	2.66E-13	2.33E-12
KIF11	1913.824	1.412275	0.27741	5.090932	3.56E-07	1.35E-06
CEP55	482.5731	1.334555	0.403412	3.308172	0.000939	0.001988
NCAPG2	1700.24	1.326956	0.272845	4.863398	1.15E-06	4.02E-06
CCNB2	795.6173	1.318156	0.33368	3.950366	7.80E-05	0.0002
CDCA8	692.7212	1.238473	0.308255	4.017694	5.88E-05	0.000154

### Effect of CENPE Interference on Cell Cycle, Cell Apoptosis, and Ara-C Drug Sensitivity

**Figure 2:**
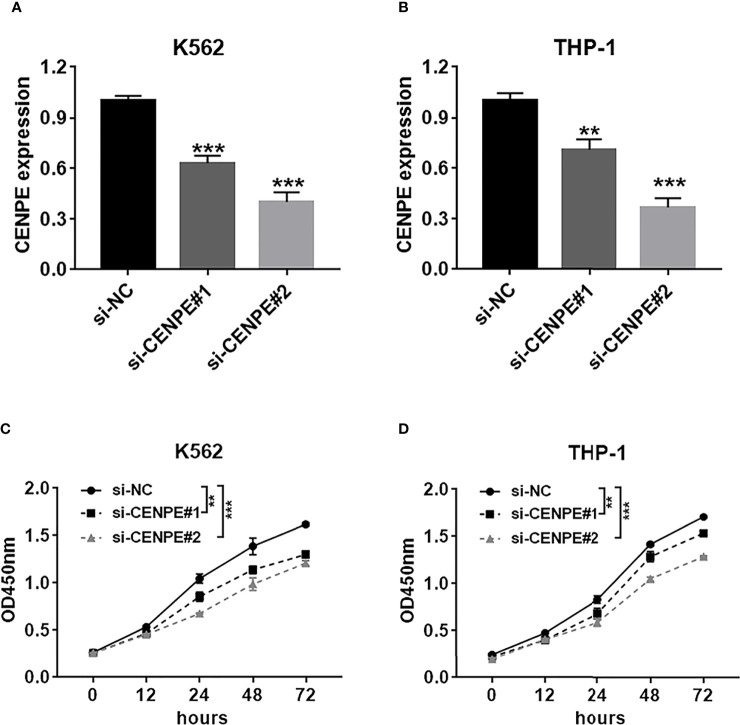
CENPE interference inhibited K562 and THP-1 cell proliferation. **(A, B)** K562 and THP-1 cells were transfected with si-NC, si-CENPE#1, or si-CENPE#2. Knockdown efficiency of CENPE in K562 and THP-1 cells was measured by RT-qPCR. **(C, D)** Cell proliferation was evaluated by CCK-8 assay, and si-CENPE significantly inhibited K562 and THP-1 cell activities compared with the si-NC group. **p < 0.01. ***p < 0.001.

**Figure 3:**
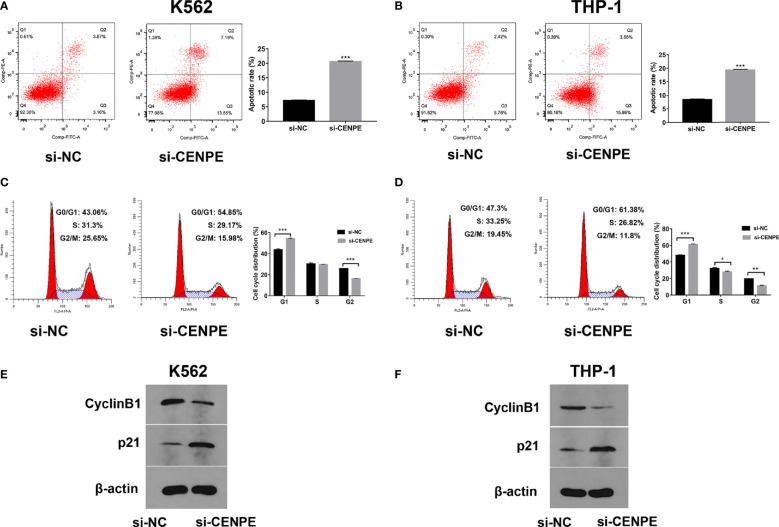
CENPE interference effected K562 and THP-1 cell apoptosis and cell cycle and drug resistance. **(A, B)** After 48 h of transfection, cell apoptosis was measured by flow cytometry. The cell apoptotic rates between si-NC and si-CENPE groups were analyzed in K562 and THP-1 cells. **(C, D)** After 48 h of transfection, cell cycle was measured by PI single-staining method. **(E, F)** After 48 h, Western blot analyzed the expression of cycle-related proteins Cyclin B1 and p21 in K562 and THP-1 cells. *p < 0.05. **p < 0.01. ***p < 0.001.

**Figure 4:**
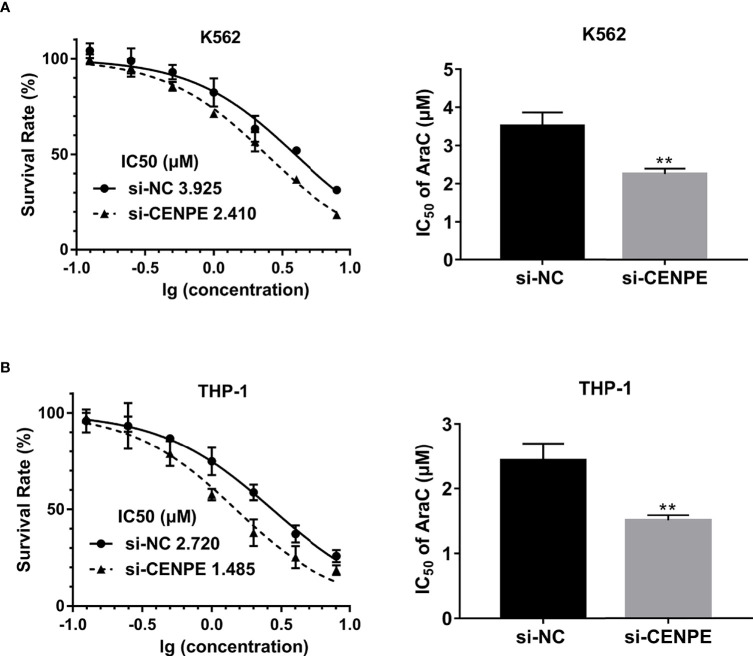
CENPE interference effected Ara-C resistance in K562 and THP-1 cells. **(A, B)** K562 and THP-1 cells were treated with ascending concentrations of Ara-C (0.125 µM, 0.25 µM, 0.5 µM, 1 µM, 2 µM, 4 µM, and 8 µM). After 48 h, IC50 values were measured and analyzed by the CCK-8 method. The experiment was independently repeated three times and statistical differences between the si-NC and si-CENPE groups were analyzed. **p < 0.01.

To further explore the functional role of CENPE in AML progression and chemoresistance, we have designed and synthesized siRNAs against CENPE (si-CENPE) and NC siRNAs (si-NC). The knockdown efficiency was analyzed and showed that si-CENPE transfection resulted in markedly reduced CENPE expression in K562 and THP-1 cells when compared with the si-NC (**Figures 2A, B**). Cell proliferation activities of K562 and THP-1 cells were analyzed by CCK-8 assay. The results showed that transfection with si-CENPE significantly inhibited K562 and THP-1 cell activities (*p* < 0.05, **Figures 2C, D**). The apoptosis of K562 and THP-1 cells after CENPE interference was analyzed by flow cytometry. The results demonstrated that CENPE interference increased the incidence of apoptosis in K562 and THP-1 cells (**Figures 3A, B**). Also, cell cycles were analyzed by PI single-staining method. The results revealed that si-CENPE transfection induced G1 phase block and reduced the number of cells of G2/M phase in K562 and THP-1 cells compared to the si-NC group (**Figures 3C, D**). Western blot was used to analyze the expression of cycle-associated proteins Cyclin B1 and p21. Compared with the si-NC group, CENPE knockdown suppressed Cyclin B1 expression and promoted p21 expression in K562 and THP-1 cells (**Figures 3E, F**), indicating that CENPE interference caused arrest and hindered the progression of the cell cycle. Moreover, Ara-C drug sensitivity after CENPE interference was detected. Following the treatment of Ara-C with different concentrations, the IC50 values were measured and analyzed by the CCK-8 method. The results showed that si-CENPE transfection reduced the IC50 values of K562 and THP-1 cells and led to enhanced sensitivity of Ara-C compared to the si-NC group (**Figures 4A, B**). In conclusion, the proliferation of myeloid leukemia cells was inhibited and chemoresistance was reversed after knocking down the expression of CENPE.

### CENPE Expression Was Highly Correlated With RBP LIN28A

**Figure 5:**
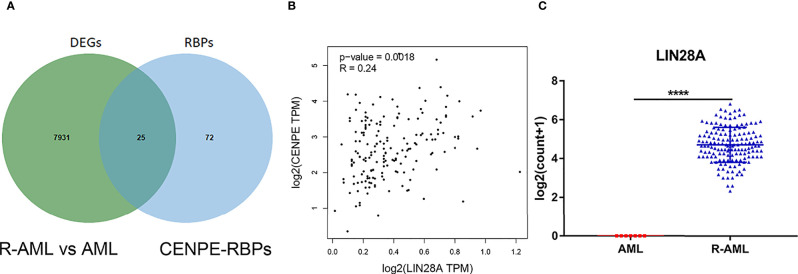
CENPE expression was highly correlated with RBP LIN28A. **(A)** Starbase database was used to screen out 25 RBPs, which might bind to CENPE and were differentially expressed in R-AML. **(B)** CENPE expression was strongly related to RBP LIN28A (*r* = 0.24; *p* < 0.05). **(C)** LIN28A gene expression were higher in the 151 R-AML whole blood samples from the TCGA database versus 7 primary AML samples from the GEO database. ****p < 0.0001.

Starbase database was used to predict the RBPs, which might bind to CENPE. Combined with the DEGs screened by TCGA R-AML patients, 25 RBPs that were differentially expressed in R-AML and might interact with CENPE were screened (**Figure 5A** and **Table 2**). The correlation between the expression of each of the above RBPs in AML and CENPE expression was analyzed using the GEPIA database (**Table 2**). LIN28A was among the top five RBPs that most correlated with CENPE in AML. CENPE expression was shown to be highly correlated with RBP LIN28A (*r* = 0.24; *p* < 0.05) (**Figure 5B**). Taking into consideration the crucial modulatory effects of LIN28A in oncogenes and mRNAs and the potential roles of LIN28A on cell cycle-related genes. LIN28A was selected for further study. LIN28A gene expression levels were analyzed in the 151 R-AML whole blood samples from the TCGA database and 7 primary AML samples from the GEO database. Our preliminary analysis revealed that the expression of LIN28A was dramatically increased in R-AML patients when compared with primary AML patients (*p* < 0.05, **Figure 5C**), which means patients with high expression of LIN28A are more likely to relapse. Therefore, we further explored the modulatory role of LIN28A on CENPE.

**Table 2:** The correlation between CENPE and RBPs.

Gene	Correlation coefficient	*p*-value
VIM	−0.24	0.0016
LIN28A	0.24	0.0018
MSI1	0.22	0.0041
SLTM	0.17	0.026
FMR1	0.17	0.029
FBL	−0.16	0.038
ACIN1	−0.16	0.04
TARDBP	0.15	0.047
SRSF3	0.15	0.05
HNRNPK	0.15	0.054
HNRNPC	0.15	0.056
U2AF1	0.14	0.063
TNRC6A	0.12	0.11
NPM1	0.12	0.12
RBM5	−0.098	0.2
SRSF9	−0.098	0.2
HNRNPA1	0.091	0.23
CNBP	−0.065	0.4
LARP4B	0.065	0.4
EIF4A3	0.056	0.46
YWHAG	0.042	0.58
IGF2BP3	−0.019	0.8
SBDS	−0.0096	0.9
KHDRBS2	0.0032	0.97
KHDRBS3	−0.023	0.77

### LIN28A Effected CENPE Expression and mRNA Stability

**Figure 6:**
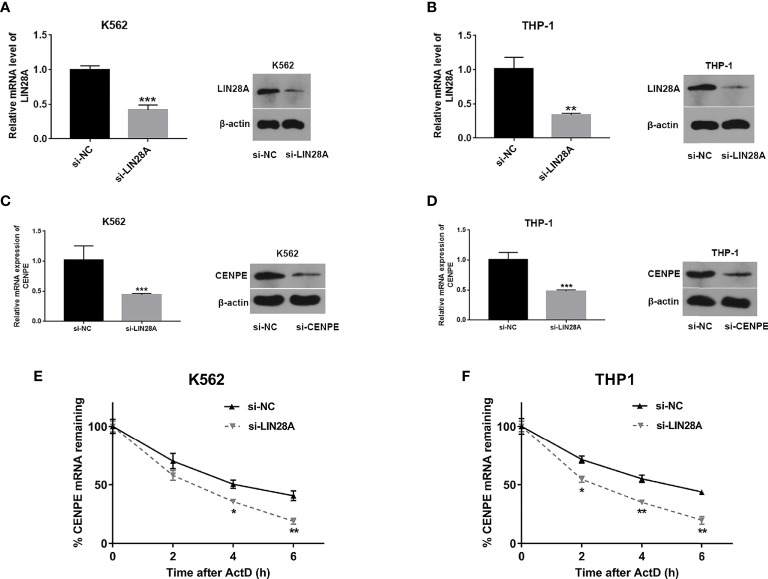
LIN28A effected CENPE expression and mRNA stability. **(A, B)** Transfected K562 and THP-1 cells with si-NC or si-LIN28A, and LIN28A mRNA and protein were detected by RT-qPCR and Western blot 48 h after transfection. **(C, D)** Forty-eight hours after transfection of LIN28A in K562 and THP-1 cells, CENPE mRNA and protein were examined by RT-qPCR and Western blot. **(E, F)** The impact of the knockdown of LIN28A on the stability of CENPE mRNA was evaluated by the actinomycin D assay. *p < 0.05. **p < 0.01. ***p < 0.001.

Analysis of transfection efficiency revealed that the si-LIN28A group led to a significant downregulation of LIN28A levels in K562 and THP-1 cells compared to the si-NC group (**Figures 6A, B**). RT-qPCR and Western blot assays further showed that knockdown of LIN28A suppressed the CENPE mRNA and protein production in K562 and THP-1 cells (**Figures 6C, D**). The influence of the LIN28A deletion on the stability of CENPE mRNA was investigated by ActD assays. At the same time after ActD treatment, the half-lives of CENPE mRNA were dramatically shortened in K562 and THP-1 cells that were transfected with si-LIN28A in comparison with the si-NC group (**Figures 6E, F**). It indicated that LIN28A interference reduced CENPE mRNA stability. In conclusion, LIN28A inhibited the CENPE mRNA and protein production, and reduced CENPE mRNA stability in myeloid leukemia cells.

### LIN28A Interacted With CENPE by Binding to the 3’UTR Region

**Figure 7:**
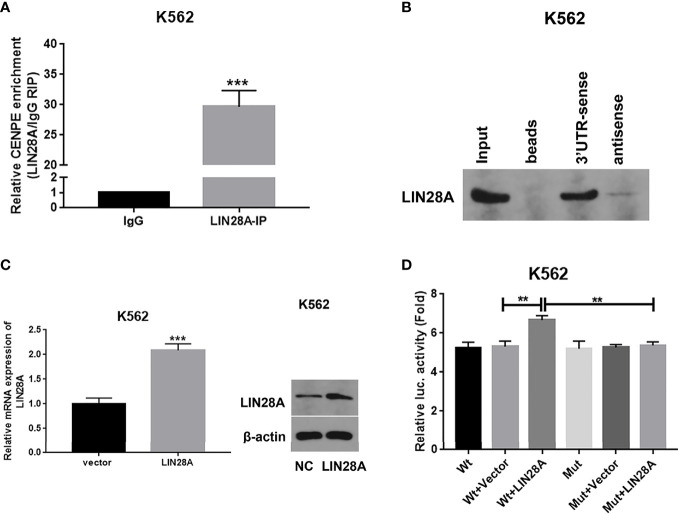
LIN28A interacted with CENPE by binding to the 3’UTR region. **(A)** CENPE mRNA enriched by IgG or LIN28A antibodies in K562 cells were detected by RIP-conjugated RT-qPCR. **(B)** RNA pull-down and Western blot assays were conducted to detect the LIN28A protein levels being pulled down by biotin sense or antisense CENPE 3’UTR. **(C)** K562 cells were transfected with empty vector or LIN28A overexpression plasmid, and LIN28 expression was detected by RT-qPCR and Western blot. **(D)** The CENPE Wt and CENPE Mut were transfected in K562 cells, including CENPE Wt+Vector, CENPE Wt+LIN28A, CENPE Mut+Vector, and CENPE Mut+LIN28A, and 48 h after cell transfection, changes in luciferase activity were measured with a luciferase activity assay kit. **p < 0.01. ***p < 0.001.

The binding capacity was investigated between LIN28A and CENPE mRNA by RIP assay. The results indicated that LIN28A antibody was able to enrich a significant amount of CENPE in K562 cells compared to the IgG group (*p* < 0.05, **Figure 7A**). Predictive analysis showed the existence of a GGAGA motif that bound to LIN28A in the CENPE 3’UTR; therefore, we hypothesized that LIN28A might impact the stability of CENPE by interacting with the CENPE 3’UTR GGAGA motif. The CENPE 3’UTR was obtained by *in vitro* transcription and labeled with a biotin synthetic probe, and we also analyzed the interaction of LIN28A with the CENPE 3’UTR by RNA pull-down assay and luciferase assay. RNA pull-down and Western blot analyses indicated that in K562 cells, LIN28A could be markedly enriched with biotinylated sense CENPE 3’UTR, whereas it could not be enriched with biotinylated antisense CENPE 3’UTR (**Figure 7B**). The LIN28A mRNA and protein levels were obviously increased in LIN28A-transfected K562 cells when compared to the Vector group (*p* < 0.05, **Figure 7C**). It indicated that the overexpression plasmid of LIN28A had a good overexpression efficiency. Wild-type (Wt) and mutant (Mut) luciferase plasmids of 100 bp upstream and downstream of the CENPE 3’UTR binding site were constructed, and CENPE Wt and CENPE Mut were transfected into K562 cells, including CENPE Wt+Vector, CENPE Wt+LIN28A, CENPE Mut+Vector, and CENPE Mut+LIN28A. Forty-eight hours after transfection, the change of luciferase activity was measured by the luciferase activity assay kit. The results revealed that the luciferase activity was remarkably stronger in the Wt group after LIN28A overexpression when compared to the Wt+Vector group (*p* < 0.05, **Figure 7C**). However, the promotion effect of LIN28A on luciferase activity in the Wt group disappeared after CENPE 3’UTR mutation (**Figure 7D**). This suggested that LIN28A can target binding to the GGAGA site of the CENPE 3’UTR.

### Interaction of LIN28A and CENPE Effected AML Cell Proliferation, Apoptosis, Cell Cycle, and Ara-C Resistance

**Figure 8:**
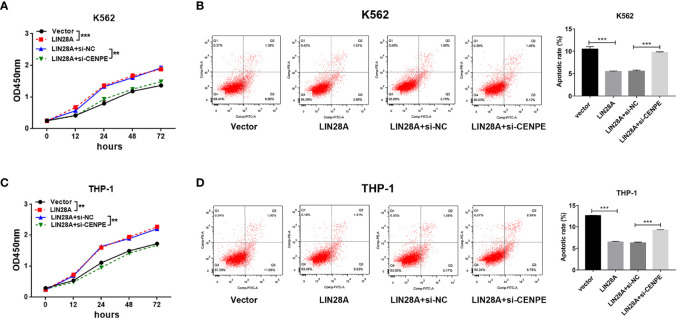
Interaction of LIN28A and CENPE effected cell proliferation and apoptosis in K562 and THP-1 cells. **(A)** CCK8 assay was used to explore the effect of LIN28A overexpression and CENPE interference on cell proliferation regulated by LIN28A overexpression of K562 cells. **(B)** After 48 h of transfection, cell apoptosis was measured by Annexin V-FITC/PI double-staining method flow cytometry in LIN28A overexpressed and CENPE interfered LIN28A overexpressed K562 cells. The cell apoptotic rates were analyzed in K562 cells. **(C)** CCK8 assay was used to explore the effect of LIN28A overexpression and CENPE interference on cell proliferation regulated by LIN28A overexpression of THP-1 cells. **(D)** After 48 h of transfection, cell apoptosis was detected by Annexin V-FITC/PI double-staining method flow cytometry in LIN28A overexpressed and CENPE interfered LIN28A overexpressed THP-1 cells. The cell apoptotic rates were analyzed in THP-1 cells. **p < 0.01. ***p < 0.001.

**Figure 9:**
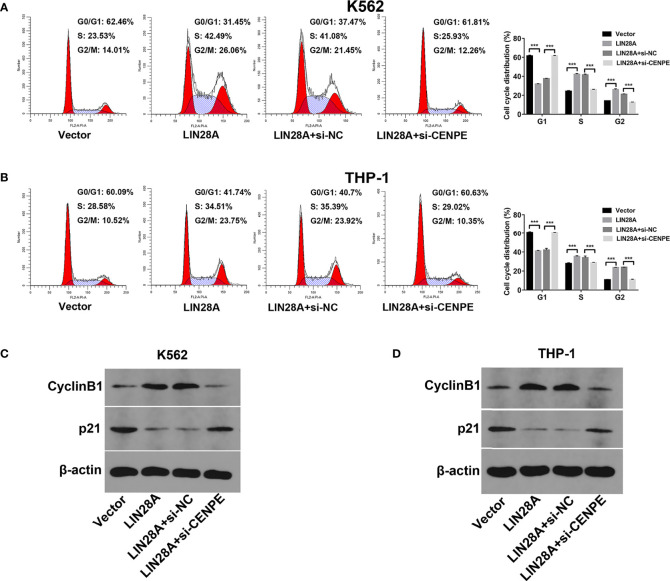
LIN28A/CENPE inhibited the cell cycle progression in K562 and THP-1 cells. **(A, B)** After 48 h of transfection, cell cycle was measured by PI single-staining method in LIN28A overexpressed and CENPE interfered LIN28A overexpressed K562 and THP-1 cells. **(C, D)** After 48 h, Western blot analyzed the expression of cycle-related proteins Cyclin B1 and p21 in LIN28A overexpressed and CENPE interfered LIN28A overexpressed K562 and THP-1 cells. ***p < 0.001.

**Figure 10:**
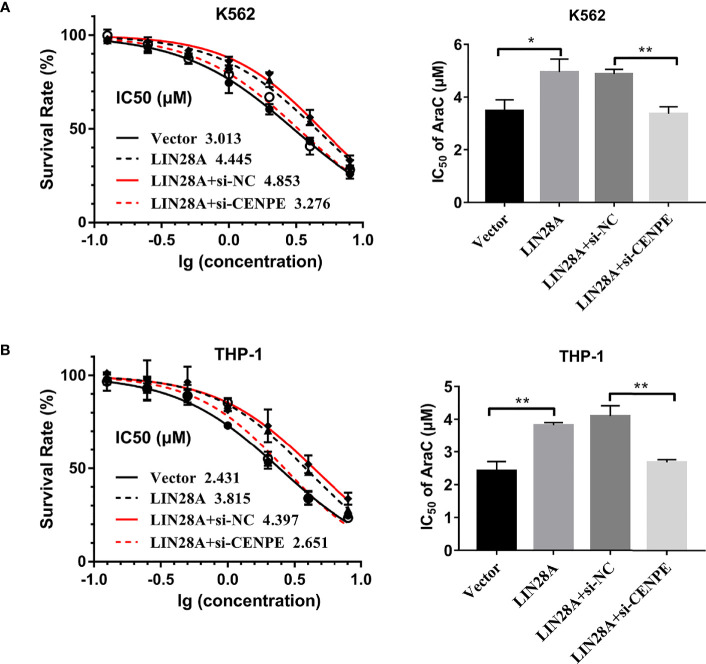
LIN28A/CENPE interaction effected Ara-C resistance in K562 and THP-1 cells. **(A, B)** LIN28A overexpressed and CENPE interfered LIN28A overexpressed K562 and THP-1 cells were treated with ascending concentrations of Ara-C (0.125 µM, 0.25 µM, 0.5 µM, 1 µM, 2 µM, 4 µM, and 8 µM). After 48 h, IC50 values were measured and analyzed by the CCK-8 method. The experiment was independently repeated three times and statistical differences were analyzed. *p < 0.05. **p < 0.01.

After LIN28A was overexpressed, the CCK-8 results revealed a significantly increased proliferation rate in K562 and THP-1 cells (*p* < 0.05, **Figures 8A, C**). The effect of CENPE interference on cell proliferation regulated by LIN28A overexpression was further analyzed. The results showed that compared with LIN28A overexpression plus si-NC group (LIN28A+si-NC), CENPE interference reversed the proliferation of K562 and THP-1 cells promoted by LIN28A overexpression (*p* < 0.05, **Figures 8A, C**). This indicated that LIN28A promoted AML cell proliferation, and CENPE interference diminished the pro-proliferative effect of LIN28A. LIN28A overexpression reduced the apoptosis rate of K562 and THP-1 cells compared with Vector (**Figures 8B, D**). Furthermore, LIN28A overexpression inhibited AML cell apoptosis, and compared with the LIN28A overexpression plus si-NC group, CENPE interference reversed the apoptosis-inhibiting ability of LIN28A overexpression (**Figures 8B, D**). In K562 and THP-1, LIN28A overexpression triggered cell cycle progression to the G2/M phase compared to the Vector group (**Figures 9A, B**). Compared with the LIN28A overexpression plus si-NC group, CENPE interference reversed the promotive effect of LIN28A overexpression on K562 and THP-1 cell cycles (**Figures 9A, B**). LIN28A overexpression induced Cyclin B1 expression and inhibited p21 expression in K562 and THP-1 cells in comparison with the Vector group (**Figures 9C, D**). Compared with the LIN28A overexpression plus si-NC group, CENPE interference reversed the regulation of Cyclin B1 and p21 expression by LIN28A overexpression (**Figures 9C, D**). Moreover, Ara-C drug sensitivity after LIN28A overexpression and CENPE interference was detected. Following the treatment of Ara-C with concentrations of 0.125 µM, 0.25 µM, 0.5 µM, 1 µM, 2 µM, 4 µM, and 8 µM in K562 and THP-1 cells, the IC50 values were measured and analyzed by the CCK-8 method. The results showed that LIN28A overexpression increased IC50 values compared to the Vector group in K562 and THP-1 cells (**Figures 10A, B**). Compared with the LIN28A overexpression plus si-NC group, CENPE interference attenuated the IC50 values of cells increased by LIN28A overexpression (**Figures 10A, B**). In conclusion, LIN28A promoted AML cell cycle progression and inhibited AML cell apoptosis, and CENPE interference repressed the cell cycle progression-promoting effect of LIN28A and facilitated apoptosis in leukemic cells. Moreover, it indicated that LIN28A enhanced drug resistance of AML cells to Ara-C, but CENPE interference reversed LIN28A-regulated Ara-C resistance in leukemic cells.

## Discussion

The prognosis for AML patients remains poor, with a 5-year survival rate of <30%, even with novel therapeutic agents (8). AML is partially triggered by dysregulated cell proliferation, which involves cell cycle modulation and DNA reparation. One mechanism of chemoresistance is related to the recognition of DNA damage by cell cycle regulators (39). Hence, inhibition of cell cycle pathways can have a synergistic impact on chemotherapy (9, 44). Previous studies have shown that mitotic regulator inhibitors, such as balaceltib and polo-like kinase-1 (PLK1), are found to be effective in combination with other chemotherapeutic agents, such as low-dose Ara-C, for the treatment of patients with leukemia, AML, myelodysplastic syndrome (MDS), and MDS-progressive AML (45–48). Therefore, targeting cell cycle regulators could be a potential therapeutic target for chemoresistant AML.

In this study, we have shown that the expression of mitosis cell cycle-related gene CENPE was notably elevated in chemoresistant AML patients compared to chemosensitive AML patients, which was in line with public data of R-AML *versus* primary AML. CENPE is a microtubule motility protein that is implicated in oncogenesis of various kinds of cancer (10, 11, 15). Knockdown of CENPE in breast cancer, prostate cancer, and neuroblastoma leads to repression of the tumor proliferation (12–14). In a study of NSCLC, CENPE was found to be highly expressed and predicted poor prognosis (16). *In vitro* studies further determined that the pro-proliferative effect of CENPE expression on lung cancer cells is modulated directly by FOXM1 *via* binding to the promoter region of CENPE (17). In leukemia, GSK923295A, which inhibited CENPE motility activity, exhibited significant remission induced anti-leukemia effect in the ALL xenografts (19). In AML, single-cell RNA-seq result showed that CENPE was correlated with higher residuals (20). In the present study, we demonstrated that CENPE was increased in chemoresistance AML patients and R-AML patients from the TCGA database. Moreover, CENPE interference significantly inhibited AML cell activity and promoted cell cycle arrest and apoptosis, which is consistent with previous findings, but whether CENPE can be involved in regulating the drug sensitivity of AML to Ara-C has not been reported. In our study, our functional analysis confirmed that CENPE interference enhanced the drug sensitivity of AML cells to Ara-C.

Given the important role of CENPE in AML progression and chemoresistance, we further explored the mechanisms of upstream regulation of CENPE. It was revealed that LIN28A was significantly correlated with CENPE expression. Highly expressed RBPs LIN28A can act as a potential oncogenic factor to promote tumorigenesis, progression, and metastasis in various human cancers (27). As in previous studies, by analyzing publicly available data, our preliminary results show that LIN28A expression is substantially increased in R-AML patients compared to primary AML patients, which indicated poor prognosis in AML. Mechanistically, LIN28A can regulate its target mRNA translation (24, 28). In papillary thyroid carcinoma cells, LIN28A interference inhibited c-myc expression, which in turn reduced cell proliferation, migration, and invasion (49). Additionally, by binding to LINC00355 or GEFT 3’UTR, LIN28A moderated LINC00355-mediated GEFT expression, increased GEFT mRNA stability, and facilitated colorectal cancer formation, development, and aggression (38). In ovarian cancer, Lin28A enriched the mRNA of RAN and HSBP1, which was negatively correlated with survival and prognosis (41). In glioma cells, the Lin28A/SNHG14/IRF6 axis is pivotal for the reprogramming of glucose metabolism and the spurring of oncogenesis, and depletion of Lin28A reduced *in vivo* xenograft tumor outgrowth and prolonged nude mice survival (42). Several studies (23–26) have also revealed the underlying role of LIN28A on cell cycle-related mRNAs. For instance, tissue microarrays identified that LIN28A expression was increased in epithelial tumors and promoted cell cycle progression by regulating CDK2, CCND1, and CDC25A in cancer cells. Moreover, it has been shown that LIN28A is involved in regulating AML cell differentiation and cycle progression (43). However, the mechanism of LIN28A in regulating cell cycle progression in chemoresistance AML is rarely studied.

In our study, LIN28A highly correlated with CENPE in R-AML. We also confirmed that LIN28A, which is upregulated in R-AML, has a predicted binding site to CENPE. RIP experiments showed that LIN28A antibody significantly enriched CENPE in K562 cells. Sequence analysis revealed that the CENPE mRNA 3’UTR contains the GGAGA motif. RNA pull-down experiments confirmed that the biotin-labeled CENPE 3’UTR positive strand could enrich a large quantity of LIN28A protein, indicating that LIN28A directly interacted with CENPE mRNA 3’UTR. Subsequently, dual luciferase reporter assay showed that the binding activity of LIN28A and CENPE mRNA 3’UTR was mediated by the GGAGA motif. In summary, LIN28A promoted CENPE mRNA expression and stability through direct binding to the GGAGA motif in the CENPE 3’UTR. More importantly, by performing functional remediation studies, we further investigated the role of LIN28A in AML development and drug resistance by affecting the stability of CENPE mRNA. The results showed that CENPE interference reduced the proliferation and cycle-promoting effects of LIN28A overexpression. In drug sensitivity assays, CENPE interference reversed the promoting effect of LIN28A on Ara-C resistance in leukemic cells.

Our findings demonstrated the underlying value of CENPE and LIN28A for the early detection of chemoresistant AML. In addition, a better understanding of the functional and molecular modulation mechanisms of LIN28A/CENPE may help provide potential therapeutic targets and synergistic agents for chemotherapy-resistant AML.

## Data Availability Statement

The datasets presented in this study can be found in online repositories. The names of the repository/repositories and accession number(s) can be found below: GEO, GSE183817.

## Ethics Statement

The studies involving human participants were reviewed and approved by the Ethics Committee of Henan Provincial People’s Hospital. Written informed consent to participate in this study was provided by the participants’ legal guardian/next of kin. Written informed consent was obtained from the individual(s), and minor(s)’ legal guardian/next of kin, for the publication of any potentially identifiable images or data included in this article.

## Author Contributions

MS, JN, XN, HG, YB, JS, and WL performed the experiments, analyzed the data, and wrote the manuscript. KS, YC, and FS contributed to the conception and design of the experiments and supervision of the study. All authors contributed to the article and approved the submitted version.

## Funding

This study was partially supported by the National Natural Science Foundation of China (No. 81971508, No. 81471589, and No. 81273259), the Health Bureau of Henan Province, P.R. China (No. 201201005), the Foundation and Frontier Research Grant of Henan Provincial Science and Technology Bureau, P.R. China (No. 142300410078), and Sansheng Chunya Funds for Young Scientists.

## Conflict of Interest

The authors declare that the research was conducted in the absence of any commercial or financial relationships that could be construed as a potential conflict of interest.

## Publisher’s Note

All claims expressed in this article are solely those of the authors and do not necessarily represent those of their affiliated organizations, or those of the publisher, the editors and the reviewers. Any product that may be evaluated in this article, or claim that may be made by its manufacturer, is not guaranteed or endorsed by the publisher.
